# Cost–utility analysis of provision of e‐cigarette starter kits for smoking cessation in emergency departments: An economic evaluation of a randomized controlled trial

**DOI:** 10.1111/add.16698

**Published:** 2024-10-31

**Authors:** Jinshuo Li, Qi Wu, Steve Parrott, Ian Pope, Lucy V. Clark, Allan Clark, Emma Ward, Pippa Belderson, Susan Stirling, Timothy J. Coats, Linda Bauld, Richard Holland, Sarah Gentry, Sanjay Agrawal, Benjamin M. Bloom, Adrian Boyle, Alasdair Gray, M. Geraint Morris, Jonathan Livingstone‐Banks, Caitlin Notley

**Affiliations:** ^1^ Department of Health Sciences University of York York UK; ^2^ Norfolk and Norwich University Hospital Norwich UK; ^3^ Norwich Clinical Trials Unit, Norwich Medical School University of East Anglia Norwich UK; ^4^ Norwich Medical School University of East Anglia Norwich UK; ^5^ Department of Cardiovascular Sciences University of Leicester Leicester UK; ^6^ Usher Institute and SPECTRUM Consortium, College of Medicine University of Edinburgh UK; ^7^ Medical School University of Leicester Leicester UK; ^8^ University Hospitals of Leicester NHS Trust Leicester UK; ^9^ Royal London Hospital, Barts NHS Trust London UK; ^10^ Addenbrookes Hospital Cambridge University Hospitals Foundation Trust Cambridge UK; ^11^ Royal Infirmary Edinburgh, NHS Lothian Edinburgh UK; ^12^ Homerton University Hospital NHS Trust London UK; ^13^ Nuffield Department of Primary Care Health Sciences Oxford UK

**Keywords:** Brief intervention, cost‐effectiveness, e‐cigarette, economic evaluation, emergency department, life‐time modelling, Markov model, randomized controlled trial, smoking cessation

## Abstract

**Aims:**

To assess the cost‐effectiveness of the Cessation of Smoking Trial in Emergency Department (COSTED) intervention compared with signposting to local stop smoking service (SSS) from the National Health Service (NHS) and personal social services (PSS) perspective.

**Design, setting and participants:**

This was a two‐group, multi‐centre, pragmatic, individually randomized controlled trial set in six Emergency Departments (EDs) in urban and rural areas in the United Kingdom. Adult (≥ 18 years) daily smokers (at least one cigarette or equivalent per day) but not daily e‐cigarette users, with carbon monoxide reading ≥ 8 parts per million, attending the ED (*n* = 972) were included. The intervention consisted of provision of an e‐cigarette starter kit plus brief smoking cessation advice and referral to a local SSS. Control was an information card on how to access local SSS.

**Measurements:**

Intervention costs included costs of training and delivery. Control costs included costs of printing information cards. Costs of smoking cessation and health‐care services were estimated based on quantities reported by participants and unit costs extracted from secondary sources. The effects were measured by quality‐adjusted life years (QALYs) derived from EQ‐5D‐5L. Other outcomes were smoking cessation measures. The primary outcome was incremental cost‐effectiveness ratio (ICER), which was calculated by dividing the difference in costs by the difference in QALYs between groups.

**Findings:**

The mean intervention costs were £48 [standard error (SE) = £0] per participant and the mean control costs were £0.2 (SE = £0) per participant. Using regression estimates, total costs were £31 [95% confidence interval (CI) = –£341 to £283] higher and 6‐month QALYs were 0.004 (95% CI = –0.004 to 0.014) higher in the intervention group than in the control group. The ICER was calculated at £7750 (probability of cost‐effective at range £20 000–30 000: 72.2–76.5%).

**Conclusions:**

The UK Cessation of Smoking Trial in Emergency Department (COSTED) intervention (provision of an e‐cigarette starter kit plus brief smoking cessation advice) was cost‐effective compared with signposting to local stop smoking services under the current recommendations of the maximum acceptable thresholds.

## INTRODUCTION

The government set an objective for England in 2019 that the smoking prevalence is to be reduced to 5% or lower by 2030 [1]. In 2022, 12.7% of adult population in England smoked cigarettes, which was the lowest figure since 2011, but still would miss the target for 2030 without further action [2, 3]. In the meantime, smoking continues to cost the NHS England £3 billion a year [4].

In April 2023, a national ‘Swap to Stop’ scheme was announced, offering a free vaping starter kit to a million smokers throughout England in partnership with Stop Smoking Service (SSS) [4]. However, the number of people accessing SSS has seen a huge decline since 2012 [4]. Emergency departments (EDs) routinely see a large volume of patients [5], and these patients are more likely to be smoking [6]. Previously, brief advice and nicotine replacement therapy (NRT) have been identified as efficacious in the ED settings [7], but the same could not be said for vaping.

To determine clinical and cost‐effectiveness, the Cessation of Smoking Trial in the Emergency Department (COSTED) was conducted to compare the provision of an e‐cigarette starter kit plus brief smoking cessation advice and referral to local SSS (intervention group), with signposting to local SSS (control group) in EDs [8]. The 6‐month biochemically verified abstinence rate was 7.2% in the intervention group and 4.1% in the control group [relative risk (RR) = 1.76, 95% confidence interval (CI) = 1.03–3.01, *P* = 0.038] [9]. This article presents the economic evaluation conducted alongside the trial to determine the cost‐effectiveness from the UK National Health Service (NHS) and personal social service (PSS) perspective.

## METHOD

### Trial design

The COSTED trial was a two‐group, multi‐centre, pragmatic individually randomized controlled trial conducted in six UK EDs. Participants were eligible if they were adults (aged ≥ 18 years), self‐reporting daily smoking at least one cigarette verified by a carbon monoxide (CO) reading of ≥ 8 parts per million (p.p.m.), and attending the ED for medical treatment or accompanying a patient attending the ED. Those who required immediate medical treatment, were in police custody, had a known history of allergy to nicotine, were currently using an e‐cigarette daily or did not have the capacity to consent, were excluded. If the patient and accompanying person were both eligible and consented to participate, the accompanying person was assigned to the same group to which the patient was randomized. If only one of them was eligible and consented, the consented person was randomized [8]. This procedure generated two samples: (1) the randomized participants; and (2) a broader sample including those non‐randomized accompanying persons.

Participants in the intervention group were offered an e‐cigarette starter kit plus brief smoking cessation advice and referral to local SSS. Participants in the control group were signposted to local SSS via a printed information card.

The randomization was carried out on 1:1 ratio using a blocked design, stratified by site. The primary end‐point was 6 months post‐randomization, with smoking status also collected at 1 and 3 months post‐randomization.

### Data collection

#### Costs

All monetary values are presented in 2021/22 pounds sterling.

##### Treatment costs

Intervention costs included staff training, CO monitors, e‐cigarette starter kits and intervention delivery.

Training consisted of National Centre for Smoking Cessation and Training (NCSCT) e‐learning (7.5 hours), one bespoke session for the intervention (3 hours) and one generic Smokefree Norfolk level 2 adviser training (2 hours). All training was delivered online. The bespoke session was delivered by two members of the research team. The Smokefree Norfolk training was delivered by two stop smoking advisers. The costs of staff time were valued using staff's respective salary plus salary on‐costs. The hourly costs of the two research team members were £29.50 and £32.28, respectively. The hourly costs of stop smoking advisers were estimated as £18.01. Attendees were costed at band 4 hospital staff, whose hourly costs were £19.06 [10]. The opportunity costs of time for training were calculated by multiplying staff hourly costs by the respective time spent.

Each site was equipped with one CO monitor costing £150 and £30‐worth of mouthpieces. Assuming a depreciation rate of 3.5% [11] over 5 years of functional life with no resale value at the end of its life and all mouthpieces consumed for the trial, the estimated costs of CO monitors and accessories during the trial period were £276.30.

The e‐cigarette starter kit (the DotPro by Liberty Flights, Clitheroe, UK) cost £7.71 for device and £15.44 for pods, including 5% bulk purchase discount and excluding 20% value added tax [11]. The opportunity costs of staff time for brief advice were calculated by multiplying the duration by band 4 hospital staff hourly costs. Participants were given a leaflet containing information on the intervention (£0.39) and a tote bag (£1.47). The printing costs of the information card in the control group were £0.20 per card.

##### Smoking cessation support and health‐care services costs

The use of smoking cessation support and health‐care services were collected via a bespoke self‐reported questionnaire as part of case report form (CRF) at baseline and 6 months (Table 1) [10, 12, 13, 14, 15, 16, 17, 18, 19, 20, 21].

**TABLE 1:** Smoking cessation support and health‐care services collected and their respective unit costs (2021/2022).

	Unit costs (2021/22)	Sources
Pharmacotherapies		
Nicotine patches	£11/pack	[12, 13]
Nicotine gums	£11/pack	
Nicotine tablets (microtab)	£16/pack	
Nicotine inhalators	£1/cartridge	[12]
Nicotine lozenges	£14/pack	
Nicotine nasal spray	£17/bottle	[12, 13]
Nicotine mouth spray	£13/bottle	
Varenicline (Champix)		
0.5 mg/1 mg 2 week treatment initiation pack	£29/pack	[12]
0.5 mg/1 mg 4 week treatment initiation pack	£55/pack	
0.5 mg tablet	£0.98/tablet	
1 mg tablet	£0.98/tablet	
Bupropion (Zyban)		
150 mg tablet	£0.70/tablet	[12]
	£41.76/pack	[13]
Smoking cessation advice		
Group session in SSS	£1/session	[14, 15]
Individual session in SSS	£9/session	
GP	£38/session	[10, 16]
Practice nurse	£8/session	
Pharmacist	£5/session	
NHS Smoking Helpline	£8/call	[10, 17, 18]
Health‐care services		
A&E attendance	£113/attendance	[19]
A&E admission	£303/admission	
Hospital outpatient	£165/appointment	
Hospital admission	£2621/episode	
Day case	£1038/case	
Ambulance convoy	£390/convoy	
GP	£38/consultation	[10]
Practice nurse	£13/consultation	[10, 20]
Prescription	£19/prescription	[21]

*Note*: Only number of individual and group sessions in SSS, and number of GP visits and hospital stays in the previous 3 months was collected at baseline due to limited time in ED settings.

Abbreviations: A&E = Accident and Emergency; ED = Emergency Department; GP = General Practitioner; NHS = National Health Service; SSS = stop smoking service.

##### Participants’ spending on nicotine replacement therapy (NRT) and e‐cigarettes

The quantities of NRT products bought by participants and prices paid for e‐cigarettes and accessories during the 6‐month trial period were collected as part of CRF. The estimated prices of NRT products (Supporting information, Table S1) were then applied to reported quantities.

#### Outcomes

##### Quality‐adjusted life years (QALYs)

EQ‐5D‐5L [22] was administered at baseline and 6 months. This consists of five domains that could be converted to a utility value and a visual analogue scale (VAS) valuing the overall health on the day, ranging from 0 (worst imaginable) to 100 (best imaginable). QALYs were derived from the utility values at baseline and 6 months, following the area under the curve approach [23].

We originally planned to use the cross‐walk mapping from EQ‐5D‐3L tariff to EQ‐5D‐5L responses [24], following the guidance from NICE on conversion of EQ‐5D‐5L utility values [25] at the time. However, updated guidance has since been published recommending a new mapping approach [11], which we followed in our analysis [26].

##### Smoking cessation outcomes

CO‐validated sustained abstinence (primary outcome of the trial) was defined as self‐reported no more than five lapses at 6 months, biochemically validated by CO reading ≤ 7 p.p.m. [27]. Self‐reported sustained abstinence was defined as no more than five lapses reported by participants at 6 months. Self‐reported 7‐day abstinence was defined as having smoked no cigarettes (not even a puff) in the past 7 days, which was collected at 1, 3 and 6 months [8].

### Analyses

#### Missing data

Missing values on all smoking cessation outcomes were considered as not abstinent [27]. Missing data on other variables were handled following the methods proposed by Faria *et al*. [28]. Missing values in baseline covariates were imputed using the mean value of the variable of the full sample, as these were assumed unrelated to the treatments. Missing values in follow‐up variables were dealt with using the multiple imputation chained equation method, following Rubin's rule and assuming missing at random (MAR) [29]. The imputation model included all variables necessary to the analysis or associated with missingness, which were identified by univariate logistic regression or χ^2^ test. Outcome variables were imputed using predictive mean matching, with the 10 closest neighbouring values to draw from [30]. The imputation was performed separately by randomized groups and stratified by sites, augmented for perfect prediction. The number of imputations was set as approximately the highest percentage figure of missing data [30]. Unless otherwise specified, all analyses were performed on the multiple imputed data set.

#### Primary analysis

Using costs of treatments and smoking cessation support during the 6 months and CO‐validated 6‐month sustained abstinence, cessation costs per abstainer were calculated for both groups, together with incremental costs per additional abstainer. Total costs included costs of treatments, smoking cessation support and health‐care services. An incremental cost–utility analysis (CUA) was conducted using total costs and QALYs during the 6‐month period. No discount was applied. Incremental costs and QALYs were estimated using generalized linear regression models, adjusting for demographic covariates, costs of smoking cessation and health‐care services before baseline and EQ‐5D‐5L utility at baseline, respectively, and ED site. Incremental costs were divided by incremental QALYs to generate an incremental cost‐effectiveness ratio (ICER) when the intervention group resulted in both higher costs and higher QALYs than the control group. The ICER was compared with the maximum acceptable thresholds of £20 000–30 000 per QALY gain [11].

Uncertainty was assessed using a non‐parametric bootstrap re‐sampling technique [31]. The bootstrap and multiple imputation generated 5000 pairs of estimates of incremental costs and effects to construct the 95% confidence intervals (CIs) for incremental costs and effects. A cost‐effectiveness plane (CEP) was plotted to demonstrate the uncertainty of the ICER. Cost‐effectiveness acceptability curves (CEACs) [9] were plotted to show the probability that the intervention is cost‐effective at different thresholds.

##### Sensitivity analyses

Self‐reported sustained smoking abstinence at 6 months was adopted to examine the impact of missing CO readings. Self‐reported 7‐day point prevalence abstinence at follow‐ups was adopted to estimate costs per quitter at different time‐points and provide wider comparability with existing literatures.

To assess the impact of imputing missing data, a complete case analysis was conducted among the participants who had complete costs and QALYs at baseline and 6‐month follow‐up and smoking status at 6 months, following the same method as the primary analysis. Sensitivity analyses using pattern mixture modelling were conducted to examine the MAR assumption for multiple imputation methods [28]. Under the missing not at random (MNAR) assumption, it was assumed that those who had missing outcome measures at 6 months were either in higher need of health‐care services or experiencing worse health, or both at the same time. To examine how MNAR assumptions would affect the results, the incremental estimates were re‐estimated based on: (1) imputed costs increased by 10, 20 and 30%; (2) imputed utility at 6 months reduced by 10, 20 and 30%; and (3) the combination of (1) and (2).

#### Secondary analyses

#### Participants’ spending on NRT and e‐cigarettes

Difference in spending was estimated using a generalized linear regression model, adjusting for demographic covariates, spending on e‐cigarette at baseline and ED site. The uncertainty was presented using bootstrapped 95% CI.

##### Analysis on the broader sample

An incremental CUA was conducted following the same approach as the primary analysis, but on the broader sample, including the non‐randomized accompanying individuals.

##### Long‐term cost‐effectiveness projection

As improved health and health‐care cost‐saving resulting from reduced risks of developing smoking‐related diseases (SRDs) due to quitting are likely to be reflected in the long term [32], the time horizon of 6 months may fail to capture the full benefit of the intervention [33]. A Markov model [34] was adapted to project life‐time impacts of the intervention compared to control. The model runs on 1‐year cycle transitioning between smokers, ex‐smokers and deaths, until a cohort of 1000 smokers reach 90 years or death (Supporting information Figure S1). The transition probabilities were estimated based on mortalities [35], RRs of death among smokers [32], natural quit rate and relapse rates [36, 37] (Supporting information Table S2). Smoking‐attributable costs (SACs) were estimated following the smoking‐attributable proportion approach [38], based on RRs of SRDs [39], hospital admission episodes of SRDs [40] and matching inpatient costs by Hospital Resources Grouper [41], inflated to the analysis year [10]. QALYs were estimated based on age, gender and smoking status [42]. A discount rate of 3.5% per annum was applied to all costs and QALYs [11]. A probabilistic sensitivity analysis was conducted using Monte Carlo simulation. Detailed description of the model and parameters are presented in the Supporting information, Long‐term model description.

All analyses were undertaken following the pre‐registered analysis plan (https://osf.io/gevch). All analyses adopted the NHS and PSS perspective, as per National Institute for health and Care Excellence (NICE) guidance [11], except for participants’ spending on smoking cessation aids. Participants were analysed in their allocated groups, following the intention‐to‐treat principle. The long‐term model projection was performed in Microsoft Excel. Other analyses were performed in StataMP version 18.0.

## RESULTS

From January to August 2022, 972 participants were randomized to control group (*n* = 488) or intervention group (*n* = 484). The mean age was 40.5 years old [standard deviation (SD) = 13.7] in the control group, with 38.3% (187/488) female and 40.5 (SD = 13.6) years old in the intervention group, with 37.6% (182/484) female (Supporting information, Sample characteristics, Table S3).

### Treatment costs

#### Intervention costs

The training costs for the COSTED intervention were estimated at £6690, equalling £14 per participant. One participant in the intervention group did not receive the e‐cigarette. Five participants were not referred to local SSSs. The mean duration of intervention delivery was 25.7 minutes (SD = 7.3 minutes). The mean intervention costs were £48 (SD = £3) per participant (Table 2).

**TABLE 2:** Breakdown of COSTED intervention costs (*n* = 484).

Cost item	Unit cost (2022/23)	Description	Costs
Staff training			
Research team			
Band 8 Band 7	£32.28/hour £29.50/hour	Bespoke online training 3 hours × (£32.28 + £29.50)	£185
Stop smoking adviser	£18.01/hour	Generic smoke‐free Norfolk level 2 training 2 hours × £18.01 × 2 people	£72
Trainees (band 4 hospital staff)	£19.06/hour	(NCSCT e‐learning 7.5 hours + bespoke online training 3 hours + generic smoke‐free Norfolk level 2 training 2 hours) × £19.06 × 27 people	£6433
Total training costs			£6690
Average training costs per participant			£14
Intervention delivery			
CO monitors	£150/device £30‐worth of mouthpieces	£150 per device/site × 6 sites over 6 months, with 5 years’ operating life and 3.5% depreciation rate + £30/site × 6 sites = £276.30	£0.57 per participant
E‐cigarette starter kits	£23.15/kit	£7.71 for device + £15.44 for podsOne participant did not receive	£23.10 (SD £1.05) per participant
Information leaflets	£0.39/leaflet	Invoice payments £472/1200 leaflets	£0.39 per participant
Tote bags	£1.47/bag	Invoice payments £953.40/650 bags	£1.47 per participant
Brief advice	£19.06/hour	Mean duration 25.7 minutes (SD = 7.3) per participant × £19.06/hour (band 4 hospital staff) /60 minutes	£8.17 (SD £2.33) per participant
Average intervention delivery costs per participant			£34 (SD £3)
Mean intervention costs per participant			£48 (SD £3)

Abbreviations: CO = carbon monoxide; COSTED = Cessation of Smoking Trial in Emergency Department; NCSCT = National Centre for Smoking Cessation and Training; SD = standard deviation.

#### Control costs

All 488 participants in the control group were given the information card, making the mean control costs £0.20 (SD = £0) per participant.

### Missing data

The follow‐up rate at 6 months was 65.0% (317/488) in the control group and 72.5% (351/484) in the intervention group (χ^2^ = 6.4642, *P* = 0.011). Most missing values were due to lost‐to‐follow up rather than individual items missing, leading to a higher level of missing values in the control group than in the intervention group (Supporting information, Table S4). After examining the missing data (Supporting information, Tables S5–S7), the multiple imputation model included the baseline covariates [age, gender, Fagerström test of cigarette dependence (FTCD), other smokers in the household, reason for ED attendance and ED site], outcome measures at baseline and 6 months (costs of smoking cessation advice, spending on e‐cigarettes, EQ‐5D‐5L utility and VAS), costs of health‐care services at baseline and outcome measures at 6 months [CO‐validated abstinence, costs of pharmacotherapies (NRT, varenicline and bupropion), costs of primary care services, costs of secondary care services and spending on NRT]. The number of multiple imputations was set as 45. For detailed information please see Supporting information, Missing data.

### Primary analysis

The CO‐validated 6‐month sustained abstinence rate was 4.1% (20/488, SE = 0.9%) in the control group and 7.2% (35/484, SE = 1.2%) in the intervention group. The mean costs of control were £0.2 (SE = £0) and that of intervention was £48 (SE = £0). The mean control and intervention costs per CO‐validated sustained abstainer were estimated at £5 (SE = £1) and £657 (SE = £107), respectively. In addition, costs of smoking cessation help that was not provided by our study were £24 (SE = £4) per participant in the control group and £16 (SE = £4) per participant in the intervention group during the 6‐month follow‐up. Including these costs, the mean costs per CO‐validated sustained abstainer increased to £597 (SE = £164) in the control group and £876 (SE = £151) in the intervention group. The incremental costs per additional abstainer for the intervention compared to control were £1255 (95% CI = £550–6090).

The mean total costs per participant during the 6‐month period were £1651 (SE = £276) in the control group and £1408 (SE = £171) in the intervention group (Table 3). The mean QALYs per participant during the 6‐month period were 0.290 (SE = 0.007) in the control group and 0.303 (SE = 0.006) in the intervention group.

**TABLE 3:** Results of mean (SE) costs and QALYs of primary analysis, complete case analysis and analysis of broader sample.

Mean (SE)	Primary analysis based on imputed data set		Complete case analysis		Analysis of broader sample	
	Control (*n* = 488)	Intervention (*n* = 484)	Control (*n* = 502)	Intervention (*n* = 505)	Control (*n* = 502)	Intervention (*n* = 505)
Baseline costs						
Costs of smoking cessation and health‐care services	£710 (96)	£631 (110)	£529 (99)	£333 (69)	£691 (94)	£613 (105)
Costs during 6‐month period						
Costs of control/intervention	£0.2 (0)	£48 (0)	£0.2 (0)	£48 (0)	£0.2 (0)	£47 (0)
Costs of smoking cessation	£24 (4)	£16 (4)	£27 (5)	£17 (5)	£24 (4)	£15 (3)
Costs of primary care services	£133 (12)	£127 (12)	£132 (14)	£130 (14)	£132 (12)	£128 (11)
Costs of secondary care services	£1494 (274)	£1218 (169)	£1403 (290)	£1101 (170)	£1427 (263)	£1153 (147)
Total costs	£1651 (276)	£1408 (171)	£1561 (293)	£1295 (174)	£1584 (265)	£1343 (149)
EQ‐5D‐5L utility						
Baseline	0.527 (0.015)	0.550 (0.015)	0.564 (0.018)	0.552 (0.019)	0.534 (0.015)	0.560 (0.015)
6 months	0.634 (0.017)	0.660 (0.016)	0.654 (0.019)	0.662 (0.019)	0.639 (0.017)	0.660 (0.017)
QALYs	0.290 (0.007)	0.303 (0.006)	0.305 (0.008)	0.304 (0.008)	0.293 (0.007)	0.305 (0.006)

Abbreviations: SE = standard error; QALYs = quality‐adjusted life years.

**FIGURE 1:**
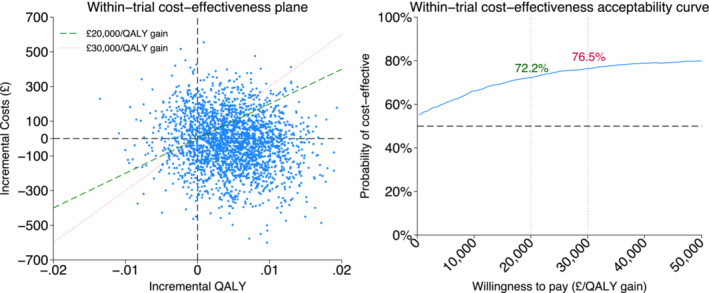
Cost‐effectiveness plane and cost‐effectiveness acceptability curve of primary analysis.

After adjusting for baseline covariates [age, gender, reason for ED attendance, FTCD, if other smoker(s) in household, and health‐care costs in the previous 3 months as fixed effects and ED site as random effect], the mean total costs in the intervention group were £31 (95% CI = –£341 to £283) higher than in the control group. After adjusting for baseline covariates [age, reason for ED attendance, FTCD, if other smoker(s) in household and EQ‐5D‐5L utility at baseline as fixed effects and ED site as random effect], the mean QALYs in the intervention group were 0.004 (95% CI = –0.004 to 0.014) higher than in the control group. The intervention was more costly and more effective than control, with an ICER calculated at £7750 per QALY gained. Most of the dots (representing bootstrapped ICERs) in Figure 1 (left) fell below the ICER threshold lines, indicating cost‐effective. This is further demonstrated by Figure 1 (right), where the probability of the intervention being cost‐effective between £20 000/QALY gain and £30 000/QALY gain was from 72.2 to 76.5%.

#### Sensitivity analyses

##### Other smoking cessation outcomes

**FIGURE 2:**
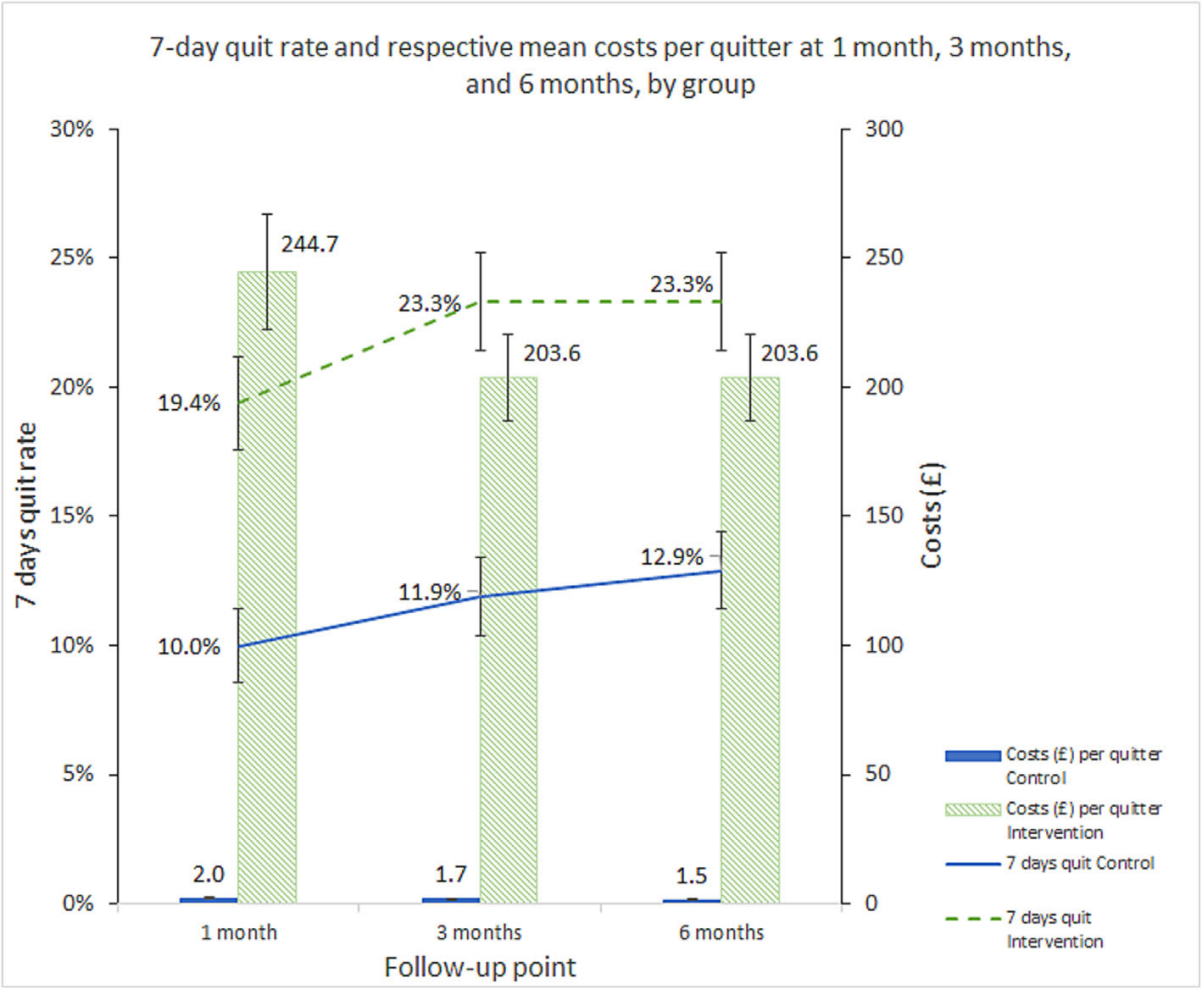
Seven‐day quit at 1, 3 and 6 months and their respective control/intervention costs per quit.

Using self‐reported outcomes, the sustained abstinence at 6 months was 13.1% (64/488, SE = 1.5%) in the control group and 25.2% (122/484, SE = 2.0%) in the intervention group, with the mean costs of control and intervention at £2 (SE = £0) and £189 (SE = £15) per abstainer, respectively. Figure 2 illustrates the 7‐day quit rate at 1, 3 and 6 months and the respective control/intervention costs per quit.

##### Complete case analysis

In total, 285 participants in the control group and 296 participants in the intervention group were included in the complete case analysis (Supporting information, Table S8). Compared to the primary analysis, complete cases in both groups appeared healthier and incurred lower costs (Table 3). The adjusted incremental analysis showed that the intervention was less costly but more effective than control, with higher uncertainty surrounding both estimates (Table 4). Supporting information, Figure S2 illustrates this increased uncertainty, but the intervention remained to be probably cost‐effective.

**TABLE 4:** Adjusted incremental costs and QALYs using generalized linear regression models.

	Primary analysis based on imputed data set	Complete case analysis	Analysis of broader sample
Adjusted incremental values (95% CI)			
Adjusted incremental costs	£31 (−£341, £283)	‐£43 (−£559, £278)	£28 (−£322, £319)
Adjusted incremental QALYs	0.004 (−0.004, 0.014)	0.002 (−0.010, 0.013)	0.003 (−0.005, 0.013)
ICER			
	£7750 per QALY gained (for uncertainty please see Figure 1)	Intervention was less costly but more effective (for uncertainty please see Supporting information, Figure S2)	£9333 per QALY gained (for uncertainty please see Supporting information, Figure S4)

Abbreviations: ICER = incremental cost‐effectiveness ratio; CI = confidence interval; QALYs = quality‐adjusted life years.

##### Analysis under MNAR assumptions

Scenarios 1 and 2 showed that adjusted incremental costs decreased with the increase of imputed costs and adjusted incremental QALYs increased with the decrease of imputed utilities (Supporting information, Table S9). Scenario 3 reported highest ICER at £5217/QALY gain when both changed by 10% and lowest ICER at £1765/QALY gain when both changed by 30% (Supporting information, Figure S3).

### Secondary analyses

#### Participants’ spending on NRT and e‐cigarettes

After adjusting for baseline covariates (age, gender, reason for ED attendance, deprivation index, FTCD, spending on e‐cigarettes in the 3 months before baseline, and ED site), the mean spending on smoking cessation aids in the intervention group was £45 (95% CI = £32–£63) higher than in the control group (Supporting information, Table S10).

#### Analysis of the broader sample

Thirty‐five accompanying individuals were allocated alongside randomized participants, with 14 (nine female) to control group and 21 (13 female) to intervention group. Table 3 shows slightly lower costs and higher QALYs in the broader sample. The adjusted incremental values were similar to those of primary analysis (Table 4). Supporting information, Figure S4 illustrates the reduced probability of the intervention being cost‐effective with these non‐randomized participants included (£20 000–30 000: 61.0–64.8%).

#### Long‐term projection

**FIGURE 3:**
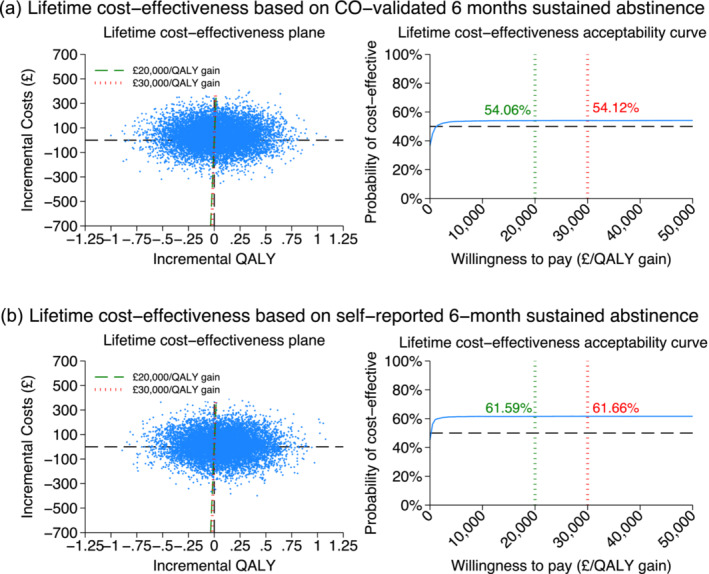
Life‐time cost‐effectiveness plane and cost‐effectiveness acceptability curve estimated by model projection.

Supporting information, Table S11 presents the input parameters estimated from the trial results. The estimated mean life‐time SACs and QALY gains of control and intervention were similar (Table 5). Compared to control, the intervention was £32 more costly per person, but 0.029 QALYs more effective. The life‐time ICER was estimated at £1131 per QALY gained. Increasing the threshold from £20 000 to £30 000 per QALY gain made little change of the probability of the intervention being cost‐effective in the long term (Figure 3).

**TABLE 5:** Results of model‐based incremental cost‐effectiveness analysis.

	Controlmean (SE)	Interventionmean (SE)	Incremental outcomesmean (95% CI)
Quit defined as CO‐validated abstinence at 6 months			
Costs	£2368 (£3)	£2400 (£3)	£32 (−£163, £231)
QALYs	25.507 (0.037)	25.535 (0.037)	0.029 (−0.489, 0.847)
ICER	£1131 per QALY gained (for uncertainty see Figure 3a)		
Quit defined as self‐reported abstinence at 6 months			
Cost	£2348 (£3)	£2361 (£3)	£13 (−£186, £207)
QALYs	25.552 (0.037)	25.626 (0.037)	0.074 (−0.746, 0.898)
ICER	£174 per QALY gained (for uncertainty see Figure 3b)		

Abbreviations: ICER = incremental cost‐effectiveness ratio; CI = confidence interval; CO = carbon monoxide; QALYs = quality‐adjusted life years.

## DISCUSSION

While the control comprised only one information card, the COSTED intervention comprised multiple components, which led to an increased cost at £48 per participant compared to £0.2 in the control group. This added complexity and cost were associated with more benefits, as the intervention resulted in a higher 6‐month CO‐validated sustained abstinence rate of 7.2%, compared to the 4.1% achieved with control. Consequently, the average costs per CO‐validated sustained abstainer at 6 months were £5 for control and £657 for the COSTED intervention. The intervention was more costly and more effective than control, with the ICER during the 6 months calculated at £7750 per QALY (probability of cost‐effectiveness between £20 000 and £30 000: 72.2–76.5%). The life‐time ICER was projected at £1131 per QALY (probability of cost‐effectiveness increased negligibly from 54.06 to 54.12% between £20 000 and £30 000).

From April to December 2022, the SSS statistics in England reported on average a cost of £797 per self‐report quitter, ranging from £24 to £6806 where data were available [43]. The definition of quit adopted by the SSS is having not smoked at all in the last 2 weeks at 4 weeks after quit date, to which the closest measure for our intervention group is 7‐day quit at 1 month. The estimated £245 per self‐reported 7‐day quitter only included the intervention costs because we did not collect smoking cessation costs outside our study at 1 month. However, considering that the mean costs of smoking cessation outside our study over 6 months were £16 (SE = £4), our costs would appear comparable with the SSS.

Miller *et al*. [44] estimated the costs of interventions for quitting smoking set in ED in two studies in the United States. The brief negotiated interviewing + NRT was the most similar intervention to COSTED, both in participant contact time (31.9 minutes) and the format (brief interview and cessation aid). After inflating the costs from 2018 to December 2022 and converting to pounds sterling (0.85 GBP = 1 USD) [45, 46], costs per quit of brief negotiated interviewing + NRT beyond usual care were approximately £1846. The definition of the quit in the studies was CO‐validated abstinence in the past 7 days at 3 months. The costs per 7‐day abstinence at 3 months of our intervention beyond our control were very similar at £1771, although this was based on self‐reported outcome.

The probability of life‐time cost‐effectiveness appears plateaued soon after willingness‐to‐pay increased from £0 per QALY. This was because, in the long term, the difference in SACs and QALYs between groups was estimated to be smaller, making the distribution of simulated pairs of incremental costs and QALYs almost symmetric surrounding the origin point, as shown on the CEPs. As willingness‐to‐pay increases, the simulated ICERs fluctuate above and below the thresholds in similar amount. The slight spread towards positive incremental QALYs resulted in probability appearing plateaued above 50%.

Participants’ spending on e‐cigarettes in the intervention group doubled the spending in the control group. This was expected and even encouraged, as the intervention only provided a starter kit of e‐cigarette and information on where to purchase further supplies. To a population with a relatively deprived socio‐economic status, the decision to encourage people who smoke to adopt e‐cigarettes to quit should not be made lightly under current policies, whereby NRT offers an alternative free‐of‐charge way of acquiring while e‐cigarettes do not. While suggestions could be made that switching from cigarettes to e‐cigarettes could save money from buying fewer cigarettes [47], this would only be true if they manage to at least cut down smoking.

The biggest strength of our study was the large sample size with broad inclusion criteria and sites in several locations across the United Kingdom with a diverse population, enabling wider generalizability and avoiding differences between groups appearing by chance, especially in terms of costs. It also shows that EDs can provide a feasible setting for opportunistic smoking cessation interventions. To our knowledge, this is the first large trial to test e‐cigarettes for smoking cessation among ED attendees.

The most concerning issue was the imbalance of follow‐up between groups, which led to higher uncertainty in estimates of the control group and, in turn, the ICER. Our analysis assumed that intervention delivery would be by existing ED staff within their working schedule. However, in a busy ED environment staff might not have the capacity. As shown by Miller *et al*. [44], there was a considerable difference between using existing staff delivering the intervention, among their multitude of other tasks, and hiring extra staff dedicated to the delivery of this intervention. Deploying staff from an in‐hospital smoking cessation department or local SSS might be potential solutions, but these rely upon the availability of those services. The results estimated in this article should not be taken for reference if extra or external staff are to be employed. In addition, our analysis did not consider the overheads, capital and other administrative costs. Inclusion of these could potentially double the intervention costs. Moreover, the long‐term effects of continued use of e‐cigarette remains unknown at the time of the analysis, and were not considered.

In conclusion, our study found that the COSTED intervention is likely to be cost‐effective compared to simple signposting. Provision of brief smoking cessation in ED should be considered with further exploration into the financial impact of different implementation approaches.

## AUTHOR CONTRIBUTIONS

**Jinshuo Li:** Data curation (supporting); formal analysis (lead); methodology (lead); visualization (lead); writing—original draft (lead). **Qi Wu:** Formal analysis (equal); methodology (equal); writing—original draft (supporting). **Steve Parrott:** Conceptualization (equal); funding acquisition (equal); methodology (supporting); supervision (equal); writing—original draft (supporting). **Ian Pope:** Conceptualization (lead); data curation (supporting); funding acquisition (lead); investigation (equal); project administration (equal); writing—original draft (supporting). **Lucy V. Clark:** Data curation (equal); investigation (supporting); project administration (lead); resources (equal); writing—original draft (supporting). **Allan Clark:** Data curation (equal); formal analysis (equal); investigation (equal); methodology (equal); writing—original draft (supporting). **Emma Ward:** Conceptualization (supporting); investigation (equal); writing—original draft (supporting). **Pippa Belderson:** Conceptualization (supporting); investigation (equal); writing—original draft (supporting). **Sue Stirling:** Data curation (equal); formal analysis (equal); methodology (equal); writing—original draft (supporting). **Timothy J. Coats:** Conceptualization (supporting); investigation (supporting); methodology (supporting); writing—original draft (supporting). **Linda Bauld:** Conceptualization (supporting); investigation (supporting); methodology (supporting); writing—original draft (supporting). **Richard Holland:** Conceptualization (supporting); investigation (supporting); methodology (supporting); writing—original draft (supporting). **Sarah Gentry:** Conceptualization (supporting); investigation (supporting); methodology (supporting); writing—original draft (supporting). **Sanjay Agrawal:** Conceptualization (equal); investigation (equal); project administration (equal); writing—original draft (supporting). **Benjamin M. Bloom:** Conceptualization (supporting); investigation (equal); methodology (supporting); writing—original draft (supporting). **Adrian Boyle:** Conceptualization (supporting); investigation (equal); methodology (equal); writing—original draft (supporting). **Alasdair Gray:** Conceptualization (supporting); investigation (equal); methodology (equal); writing—original draft (supporting). **M. Geraint Morris:** Conceptualization (supporting); investigation (equal); methodology (equal); writing—original draft (supporting). **Jonathan Livingstone‐Banks:** Conceptualization (supporting); methodology (supporting); writing—original draft (supporting). **Caitlin Notley:** Conceptualization (equal); funding acquisition (equal); investigation (equal); methodology (equal); writing—original draft (supporting).

## DECLARATION OF INTERESTS

The authors declare no competing interests.

## CLINICAL TRIAL REGISTRATION

ClinicalTrial.gov NCT04854616.

## Supporting information


**Figure S1** The Markov Model structure.
**Table S2** Transition probabilities in the Markov model.
**Figure S2** Cost‐effectiveness plane and cost‐effectiveness acceptability curve of complete case analysis.
**Figure S3** Estimated ICERs under MNAR assumptions.
**Figure S4** Cost‐effectiveness plane and cost‐effectiveness acceptability curve of analysis of the broader sample.
**Table S1** Prices of NRT products over the counter.
**Table S3** Number of participants, mean age (SD) and gender proportion by reason of ED attendance and group.
**Table S4** Missing data table in randomized sample (n = 972) by group and total.
**Table S5** Association between missing at 6 months and baseline covariates in ITT sample, examined by univariate logistic regression.
**Table S6** Association between missing at 6 months and ED site and reason for ED attendance in the randomized sample (n = 972), examined by χ^2^ test.
**Table S7** Association between observed values at baseline and missing at 6 months in the randomized sample (n = 972), examined by univariate logistic regression.
**Table S8** Comparison of age, gender and reason for ED attendance between all randomized participants and those who had complete outcome measures at 6 months.
**Table S9** Results of MNAR scenarios 1) and 2).
**Table S10** Participants’ spending on smoking cessation aids over 6 months, by group.
**Table S11** Model input parameters estimated from the trial results.

## Data Availability

The protocol, consent form, statistical analysis plan, medical ethics committee approvals, training materials and other relevant study materials are available online at https://osf.io/8hbne/. Deidentified participant data will be made publicly available within 3 months at the above address.

## References

[1] Department of Health and Social Care (DHSS) . Advancing our health: prevention in the 2020s—consultation document London, UK: DHS; 2019.

[2] Office for National Statistics (ONS) . Adult smoking habits in the UK. 2022. Available at: ons.gov.uk [accessed 14 Sept 2023]

[3] Khan J . The Khan Review: making smoking obsolete London, UK: DHS; 2022.

[4] Department of Health and Social Care (DHS) . Stopping the start: our new plan to create a smokefree generation London, UK: DHS; 2023.

[5] NHS Digital . Hospital Accident and Emergency Activity, 2022–23. Hospital Accident and Emergency Activity. 2023. Available at: nhsdigital.nhs.uk. [accessed 8 July 2024]

[6] Tolmie AD , Erker R , Oyedokun T , Sullivan E , Graham T , Stempien J . Prevalence of cigarette smoking among adult emergency department patients in Canada. West J Emerg Med. 2020;21:190–197. 33207165 10.5811/westjem.2020.9.47731 PMC7673889

[7] Bernstein SL , Dziura J , Weiss J , Brooks AH , Miller T , Vickerman KA , et al. Successful optimization of tobacco dependence treatment in the emergency department: a randomized controlled trial using the multiphase optimization strategy. Ann Emerg Med. 2023;81:209–221. 36585318 10.1016/j.annemergmed.2022.08.018 PMC9868063

[8] Notley C , Clark L , Belderson P , Ward E , Clark AB , Parrott S , et al. Cessation of smoking trial in the emergency department (CoSTED): protocol for a multicentre randomised controlled trial. BMJ Open. 2023;13:e064585. 10.1136/bmjopen-2022-064585 PMC9853266 36657751

[9] Pope I , Clark LV , Clark A , Ward E , Belderson P , Stirling S , et al. Cessation of smoking trial in the emergency department (COSTED): a multicentre randomised controlled trial. Emerg Med J. 2024;41:276–282. 38531658 10.1136/emermed-2023-213824 PMC11041600

[10] Jones K.C. , Weatherly H. , Birch S. , Castelli A. , Chalkley M. , Dargan A. et al. Unit Costs of Health and Social Care 2022. 10.22024/UniKent/01.02.100519

[11] National Institute for Health and Care Excellence (NICE) . NICE health technology evaluations: the manual (PMG36). In: Process and Methods London, UK: NICE; 2022. p. 196.

[12] NHS Business Services Authority . Prescription cost Analysis—England 2021/22. Prescription Cost Analysis—England. 2022. Available at: nhsdigital.nhs.uk. [accessed 9 Jan 2023]

[13] National Institute for Health and Care Excellence (NICE) . British National Formula. London, UK: BMJ Group and Pharmaceutical Press; 2023.

[14] Glassdoor I. Stop smoking advisor salaries in United Kingdom. 2022 Available at: glassdoor.co.uk. [accessed 15 Feb 2023]

[15] National Institute for Health and Care Excellence (NICE) . Resource impact report: Tobacco: preventing uptake, promoting quitting, and treating dependence (NG209) London, UK: NICE; 2021. 36745727

[16] Curtis LA , Burns A . Unit costs of Health and Social Care 2020 Canterbury, UK: Personal Social Services Research Unit; 2020.

[17] Wu Q , Parrott S , Godfrey C , Gilbert H , Nazareth I , Leurent B , et al. Cost‐effectiveness of computer‐tailored smoking cessation advice in primary care: a randomized trial (ESCAPE). Nicotine Tobacco Res. 2014;16:270–278. 10.1093/ntr/ntt136 24084467

[18] Curtis L , Burns A . Unit costs of Health and Social Care 2016. Canterbury, UK: Personal Social Services Research Unit; 2016.

[19] NHS England , NHS Improvement . National Cost Collection 2021/22. National Cost Collection for the NHS. Available at: nhsdigital.nhs.uk; 2023. [accessed 11 April 2023]

[20] Curtis L , Burns A . Unit costs of health and social care 2015. Canterbury, UK: Personal Social Services Research Unit; 2015.

[21] NHS Business Services Authority . PD1 reports. London, UK: NHS Business Services Authority; 2023.

[22] The EuroQol Group . EQ‐5D‐5L User Guide: Basic information on how to use the EQ‐5D‐5L instrument (version 2.1). Rotterdam, the Netherlands: The EuroQol Group; 2015.

[23] Richardson G , Manca A . Calculation of quality adjusted life years in the published literature: a review of methodology and transparency. Health Econ. 2004;13:1203–1210. 15386669 10.1002/hec.901

[24] van Hout B , Janssen MF , Feng YS , Kohlmann T , Busschbach J , Golicki D , et al. Interim scoring for the EQ‐5D‐5L: mapping the EQ‐5D‐5L to EQ‐5D‐3L value sets. Value Health. 2012;15:708–715. 22867780 10.1016/j.jval.2012.02.008

[25] National Institute for Health and Care Excellence (NICE) . Position statement on use of the EQ‐5D‐5L value set for England (updated October 2019) London, UK: NICE; 2019.

[26] Hernandez AM , Pudney S , Wailoo A . Estimating the relationship between EQ‐5D‐5L and EQ‐5D‐3L: results from a UK population study. Pharmacoeconomics. 2023;41:199–207. 36449173 10.1007/s40273-022-01218-7 PMC9883358

[27] West R . Assessing smoking cessation performance in NHS stop smoking Services: the Russell Standard (clinical) London, UK: Cancer Research UK and University College; 2005.

[28] Faria R , Gomes M , Epstein D , White IR . A guide to handling missing data in cost‐effectiveness analysis conducted within randomised controlled trials. Pharmacoeconomics. 2014;32:1157–1170. 25069632 10.1007/s40273-014-0193-3 PMC4244574

[29] Rubin DB . Statistical matching using file concatenation with adjusted weights and multiple imputations. J Bus Eco Stat. 1986;4:87–94.

[30] White IR , Royston P , Wood AM . Multiple imputation using chained equations: issues and guidance for practice. Stat Med. 2011;30:377–399. 21225900 10.1002/sim.4067

[31] Briggs AH , Wonderling DE , Mooney CZ . Pulling cost‐effectiveness analysis up by its bootstraps: a non‐parametric approach to confidence interval estimation. Health Econ. 1997;6:327–340. 9285227 10.1002/(sici)1099-1050(199707)6:4<327::aid-hec282>3.0.co;2-w

[32] Doll R , Peto R , Boreham J , Sutherland I . Mortality in relation to smoking: 50 years’ observations on male British doctors. BMJ. 2004;328:1519–7455. 15213107 10.1136/bmj.38142.554479.AE PMC437139

[33] Drummond M . Methods for the economic evaluation of health care programmes Oxford, UK: Oxford University Press; 2015.

[34] Wu Q , Gilbody S , Li J , Wang HI , Parrott S . Long‐term cost‐effectiveness of smoking cessation interventions in people with mental disorders: a dynamic decision analytical model. Value Health. 2021;24:1263–1272. 34452705 10.1016/j.jval.2021.04.002 PMC8404974

[35] Office for National Statistics (ONS) . Deaths Registered in England and Wales London, UK: ONS; 2021.

[36] Hughes JR , Peters EN , Naud S . Relapse to smoking after 1 year of abstinence: a meta‐analysis. Addict Behav. 2008;33:1516–1520. 18706769 10.1016/j.addbeh.2008.05.012 PMC2577779

[37] Godfrey C. , Ali S. , Parrott S. , Pickett K. Economic model of adult smoking related costs and consequences for England. Public Health Research Consortium. Available at: http://phrc.lshtm.ac.uk/papers/PHRC_A4-06_Final_Report.pdf 2011. [accessed 18 Feb 2015]

[38] World Health Organization (WHO) . Economics of tobacco toolkit: assessment of the economic costs of smoking. Geneva, Switzerland: WHO; 2011.

[39] Royal College of Physicians of London , Tobacco Advisory G , Royal College of Physicians of London , Tobacco Advisory G , Royal College of Physicians of London . Hiding in plain sight: treating tobacco dependency in the NHS: a report. London, UK: Royal College of Physicians; 2018.

[40] Hospital Episode Statistics Analysis, Health and Social Care Information Centre . Hospital Episode Statistics: Accident and Emergency Attendances in England 2014–15 Leeds, UK, 2016: Health and Social Care Information Centre.

[41] Department of Health (DoH) . Reference costs 2015–16 London, UK: DoH; 2016.

[42] Vogl M , Wenig CM , Leidl R , Pokhrel S . Smoking and health‐related quality of life in English general population: implications for economic evaluations. BMC Public Health. 2012;12:203. 22429454 10.1186/1471-2458-12-203 PMC3352300

[43] Population Health, Clinical Audit and Specialist Care, NHS England . Statistics on NHS stop smoking services ‐ England, April 2022 to December 2022 Statistics on NHS Stop Smoking Services—England; 2023 Available at: nhsdigital.nhs.uk. [accessed 23 June 2023]

[44] Miller TR , Johnson MB , Dziura JD , Weiss J , Carpenter KM , Grau LE , et al. Cost‐effectiveness of smoking cessation approaches in emergency departments. Am J Prev Med. 2023;65:39–44. 36710199 10.1016/j.amepre.2023.01.006 PMC10293014

[45] US Bureau of Labor Statistics . CPI Inflation Calculator Washington, DC: US Bureau of Labor Statistics; 2023.

[46] International Monetary Fund (IMF) . Exchange rates selected indicators. International Financial Statistics. Washington, DC: IMF; 2023.

[47] Jackson SE , Shahab L , Kock L , West R , Brown J . Expenditure on smoking and alternative nicotine delivery products: a population survey in England. Addiction. 2019;14:2026–2036. 10.1111/add.14709 PMC6797497 31243842

