# Supplementary material for: Cost–utility analysis of provision of e‐cigarette starter kits for smoking cessation in emergency departments: An economic evaluation of a randomized controlled trial
Source: Addiction. 2024 Oct 31;120(2):368–79. doi: 10.1111/add.16698 (PMC11707313; doi:10.1111/add.16698)
Supplement: Supplementary file 1 — Figure S1 The Markov Model structure. Table S2 Transition probabilities in the Markov model. Figure S2 Cost‐effectiveness plane and cost‐effectiveness acceptability curve of complete case analysis. Figure S3 Estimated ICERs under MNAR assumptions. Figure S4 Cost‐effectiveness plane and cost‐effectiveness acceptability curve of analysis of the broader sample. Table S1 Prices of NRT products over the counter. Table S3 Number of participants, mean age (SD) and gender proportion by reason of ED attendance and group. Table S4 Missing data table in randomized sample (n = 972) by group and total. Table S5 Association between missing at 6 months and baseline covariates in ITT sample, examined by univariate logistic regression. Table S6 Association between missing at 6 months and ED site and reason for ED attendance in the randomized sample (n = 972), examined by χ2 test. Table S7 Association between observed values at baseline and missing at 6 months in the randomized sample (n = 972), examined by univariate logistic regression. Table S8 Comparison of age, gender and reason for ED attendance between all randomized participants and those who had complete outcome measures at 6 months. Table S9 Results of MNAR scenarios 1) and 2). Table S10 Participants’ spending on smoking cessation aids over 6 months, by group. Table S11 Model input parameters estimated from the trial results. [file ADD-120-368-s001.docx]

Supplementary information

Table of Contents

[Estimated prices of NRT products 2](#_Toc172033308)

[Long-term model description 2](#_Toc172033309)

[Sample characteristics 4](#_Toc172033310)

[Missing data 4](#_Toc172033311)

[Complete case analysis 8](#_Toc172033312)

[Analysis under MNAR assumptions 9](#_Toc172033313)

[Participants’ spending on NRT and e-cigarette 10](#_Toc172033314)

[Analysis of the broader sample 11](#_Toc172033315)

[Long-model input parameters 11](#_Toc172033316)

[References 12](#_Toc172033317)

# Estimated prices of NRT products

Unlike prescription costs, NRT products are sold on the open market at various package sizes and prices are set by various sellers with or without any discount. In order to provide an estimate of participants’ spending, prices were extracted from one seller (Sainsburys online groceries store) to value the quantities bought by participants. Where there were multiple package sizes available, the more commonly bought size was selected and described. Where there were multiple brands offering the same product of the same package size at different prices, the median price was used. It should be noted that these results were highly uncertain. Table S1 presents the prices used in the estimation of participants’ spending.

Table S1 Prices of NRT products over the counter

| **NRT products** | **Prices*** |
| --- | --- |
| Patches | £12/7-patch pack |
| Gums | £17/105-gum pack |
| Tablets | £19/100-tab pack |
| Inhalators | £1.3/cartridge in a 20-cartridge pack |
| Lozenges | £13/80-loz pack |
| Nasal spray | £29/bottle |
| Mouth spray | £20/bottle |

*The prices presented here were extracted from Sainsburys.co.uk online groceries store in June 2023. No offer or discount was considered.

# Long-term model description

The Markov model assumes that participants would not receive other smoking cessation interventions or aids beyond the trial period. It is a three-state model including smokers, ex-smokers, and deaths (Figure S1). It runs on one-year cycles for a 1,000 people cohort until all enter the death state or reach 90 years, which is considered lifetime. Each state is associated with corresponding age-, gender-, and smoking status-specific EQ-5D utilities (1), and smoking-attributable inpatient costs over one year. Smoking-attributable costs (SACs) were estimated using relative risks (RRs) of incidence among smokers, hospital episodes, and inpatient costs of smoking-related diseases (SRDs), inflated to the analysis year (2-5). Smoking-attributable mortality was estimated using the all-cause mortality rates obtained from the Office for National Statistics death registry (6) and RR of death among smokers (7). The model also incorporates an annual natural quit rate of 2% and a relapse rate of 10% following cessation of smoking for the first 10 years (8-10). Ex-smokers are assumed to never relapse after 10 years. The transition rates are presented in Table S2. A discount rate of 3.5% per annum was applied to costs and QALYs (11). A probabilistic sensitivity analysis (PSA) was conducted using Monte Carlo simulation to assess the uncertainty of the model parameters. Details of methods please see the published article (12).


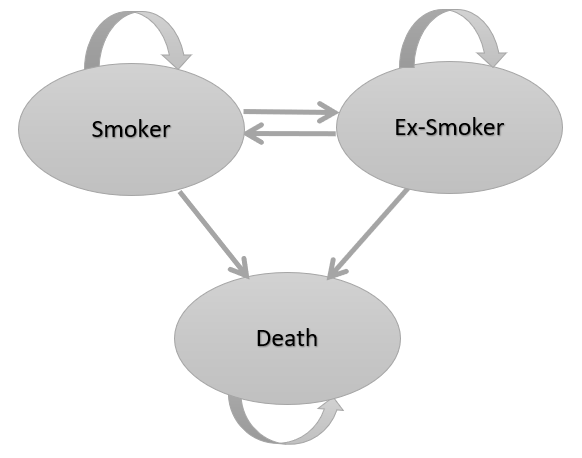


Figure S1 The Markov Model structure

Table S2 Transition probabilities in the Markov model

| **Parameters** | | **Probability** | **Sources** |
| --- | --- | --- | --- |
| **Probability of relapse for the first 10 years** | | 10% | (8, 9) |
| **Natural quit rate** | | 2% | (10) |
| **Mortality among smokers** | | | (6, 13) |
| Age group | Male | Female |  |
| 40-44 | 0.3% | 0.2% |  |
| 45-49 | 0.8% | 0.5% |  |
| 50-54 | 1.2% | 0.7% |  |
| 55-59 | 1.7% | 1.1% |  |
| 60-64 | 2.6% | 1.7% |  |
| 65-69 | 4.3% | 2.8% |  |
| 70-74 | 6.5% | 4.4% |  |
| 75-79 | 8.7% | 6.0% |  |
| 80-84 | 15.3% | 11.1% |  |
| 85-89 | 26.5% | 20.6% |  |
| **Mortality among ex-smokers** | | |  |
| 40-44 | 0.3% | 0.2% |  |
| 45-49 | 0.5% | 0.3% |  |
| 50-54 | 0.7% | 0.5% |  |
| 55-59 | 1.1% | 0.7% |  |
| 60-64 | 1.7% | 1.1% |  |
| 65-69 | 2.6% | 1.6% |  |
| 70-74 | 3.9% | 2.6% |  |
| 75-79 | 5.2% | 3.6% |  |
| 80-84 | 9.1% | 6.6% |  |
| 85-89 | 15.8% | 12.3% |  |

The model simulated a hypothetical cohort of 1,000 smokers and ex-smokers who were specified using the mean age and gender proportion of the trial sample. The abstinence rate from the primary analysis specified the proportion of smokers and ex-smokers at the beginning of the first cycle. Mean costs and mean QALYs in each group during the trial period were entered as starting point into the model. The incremental cost per additional QALY estimate was compared to maximum acceptable ICERs to establish whether the intervention offered good value for money in the use of health care resources. The current range of maximum acceptable ICERs is £20,000 - £30,000 per QALY gained (11). The uncertainty surrounding the estimated lifetime ICER was presented in a cost-effectiveness plane and CEACs based on the PSA.

# Sample characteristics

In both groups, the most common reason for attending ED was injury, followed by other medical issues (Table S3). These two reasons accounted for over half of the participants in both groups. Shortness of breath reported highest proportion of female patients in the control group, followed by abdominal pain. In the intervention group, abdominal pain reported highest proportion of female patients, followed by other reasons.

Table S3 Number of participants, mean age (SD) and gender proportion by reason of ED attendance and group

|  | **Control (n=488)** | | | **Intervention (n=484)** | | |
| --- | --- | --- | --- | --- | --- | --- |
| **Reason for attending ED** | **n (%)** | **Age Mean (SD)** | **Female (%)** | **n (%)** | **Age Mean (SD)** | **Female (%)** |
| Chest pain | 66 (13.5%) | 45.0 (13.8) | 19 (28.8%) | 54 (11.2%) | 44.7 (12.8) | 18 (33.3%) |
| Shortness of breath | 22 (4.5%) | 41.0 (13.9) | 14 (63.6%) | 22 (4.5%) | 47.1 (14.4) | 7 (31.8%) |
| Abdominal pain | 40 (8.2%) | 41.1 (13.9) | 23 (57.5%) | 40 (8.3%) | 36.9 (13.2) | 20 (50.0%) |
| Injury | 183 (37.5%) | 37.9 (13.7) | 68 (37.2%) | 184 (38.0%) | 37.6 (12.6) | 57 (31.0%) |
| Other medical issues | 120 (24.6%) | 41.5 (13.7) | 48 (40.0%) | 122 (25.2%) | 43.4 (14.1) | 54 (44.3%) |
| Mental health issues | 10 (2.1%) | 33.2 (9.2) | 3 (30.0%) | 12 (2.5%) | 39.4 (15.0) | 5 (41.7%) |
| Other | 40 (8.2%) | 43.3 (12.7) | 10 (25.0%) | 38 (7.9%) | 40.1 (13.6) | 18 (47.4%) |
| Missing | 1 (0.2%) | 35.0 (-) | 0 (-) | 0 (-) | - | - |
| Not applicable* | 6 (1.2%) | 37.3 (10.7) | 2 (33.3%) | 12 (2.5%) | 39.8 (11.9) | 3 (25.0%) |

*Not applicable indicated these were accompanying persons where the patients attending ED were illegible or did not consent.

# Missing data

Table S4 Missing data table in randomised sample (n=972) by group and total

|  | **Control (n=488)** | | **Intervention (n=484)** | | **Total (n=972)** | |
| --- | --- | --- | --- | --- | --- | --- |
| **Variables** | **n of missing** | **% of missing** | **n of missing** | **% of missing** | **n of missing** | **% of missing** |
| Age | 0 | 0% | 0 | 0% | 0 | 0% |
| Gender | 0 | 0% | 0 | 0% | 0 | 0% |
| Deprivation index | 5 | 1% | 6 | 1% | 11 | 1% |
| Smoker in same household | 0 | 0% | 0 | 0% | 0 | 0% |
| Reason for ED attendance | 1 | 0% | 0 | 0% | 1 | 0% |
| ED site | 0 | 0% | 0 | 0% | 0 | 0% |
| Intervention costs | 0 | 0% | 0 | 0% | 0 | 0% |
| Control costs | 0 | 0% | 0 | 0% | 0 | 0% |
| Baseline | | | | | | |
| FTCD | 0 | 0% | 0 | 0% | 0 | 0% |
| Have used NRT | 0 | 0% | 0 | 0% | 0 | 0% |
| Have been prescribed varenicline | 0 | 0% | 0 | 0% | 0 | 0% |
| Have been prescribed bupropion | 0 | 0% | 0 | 0% | 0 | 0% |
| Costs of smoking cessation advice | 0 | 0% | 0 | 0% | 0 | 0% |
| Costs of health care services | 0 | 0% | 0 | 0% | 0 | 0% |
| EQ-5D-5L utility | 0 | 0% | 0 | 0% | 0 | 0% |
| EQ-5D-5L VAS | 1 | 0% | 4 | 1% | 5 | 1% |
| Spending on e-cigarette | 0 | 0% | 0 | 0% | 0 | 0% |
| 6 months | | | | | | |
| Have used NRT | 185 | 38% | 161 | 33% | 346 | 36% |
| Have been prescribed varenicline | 185 | 38% | 162 | 33% | 347 | 36% |
| Have been prescribed bupropion | 185 | 38% | 162 | 33% | 347 | 36% |
| Costs of NRT prescription | 188 | 39% | 165 | 34% | 353 | 36% |
| Costs of varenicline | 185 | 38% | 163 | 34% | 348 | 36% |
| Costs of bupropion | 185 | 38% | 163 | 34% | 348 | 36% |
| Costs of smoking cessation advice | 190 | 39% | 171 | 35% | 361 | 37% |
| Costs of primary care services | 198 | 41% | 172 | 36% | 370 | 38% |
| Costs of secondary care services | 197 | 40% | 169 | 35% | 366 | 38% |
| EQ-5D-5L utility | 196 | 40% | 172 | 36% | 368 | 38% |
| EQ-5D-5L VAS | 237 | 49% | 197 | 41% | 434 | 45% |
| Spending on e-cigarette | 193 | 40% | 182 | 38% | 375 | 39% |
| Spending on NRT | 191 | 39% | 168 | 35% | 359 | 37% |

By examining the association between missing at 6 months and the baseline covariates (group allocation, age, gender, FTCD, and if smoker in household) using univariate logistic regression, the missing on all follow-up variables were found significantly associated with age (Table S5). Gender was found associated with missing on all but costs of NRT, EQ-5D-5L VAS, and spending on NRT. Group allocation was found to be only associated with missing on EQ-5D-5L VAS.

Table S5 Association between missing at 6 months and baseline covariates in ITT sample, examined by univariate logistic regression

| **n=972** | **OR (Z, p)** | | | | | |
| --- | --- | --- | --- | --- | --- | --- |
| **Missing on:** | **Allocation** | **Age** | **Gender** | **Deprivation index** | **FTCD** | **Smoker in the household** |
| Have used NRT | 0.82 (Z=-1.51, p=0.131) | **0.98 (Z=-3.27, p=0.001)** | **0.76 (Z=-1.98, p=0.048)** | 0.99 (Z=-0.53, p=0.595) | 0.99 (Z=-0.34, p=0.736) | 1.14 (Z=0.96, p=0.338) |
| Have been prescribed varenicline | 0.82 (Z=-1.44, p=0.149) | **0.98 (Z=-3.31, p=0.001)** | **0.75 (Z=-2.03, p=0.042)** | 0.98 (Z=-0.59, p=0.553) | 0.99 (Z=-0.25, p=0.805) | 1.15 (Z=1.01, p=0.311) |
| Have been prescribed bupropion | 0.82 (Z=-1.44, p=0.149) | **0.98 (Z=-3.31, p=0.001)** | **0.75 (Z=-2.03, p=0.042)** | 0.98 (Z=-0.59, p=0.553) | 0.99 (Z=-0.25, p=0.805) | 1.15 (Z=1.01, p=0.311) |
| Costs of NRT prescription | 0.83 (Z=-1.44, p=0.151) | **0.98 (Z=-3.23, p=0.001)** | 0.77 (Z=-1.92, p=0.054) | 0.98 (Z=-0.66, p=0.511) | 1.00 (Z=0.06, p=0.951) | 1.12 (Z=0.80, p=0.423) |
| Costs of varenicline | 0.83 (Z=-1.38, p=0.169) | **0.98 (Z=-3.20, p=0.001)** | **0.75 (Z=-2.08, p=0.037)** | 0.98 (Z=-0.63, p=0.528) | 0.99 (Z=-0.19, p=0.852) | 1.14 (Z=0.93, p=0.352) |
| Costs of bupropion | 0.83 (Z=-1.38, p=0.169) | **0.98 (Z=-3.29, p=0.001)** | **0.75 (Z=-2.08, p=0.037)** | 0.99 (Z=-0.50, p=0.617) | 0.99 (Z=0.27, p=0.785) | 1.14 (Z=0.93, p=0.352) |
| Costs of smoking cessation advice | 0.86 (Z=-1.16, p=0.245) | **0.98 (Z=-3.32, p=0.001)** | **0.72 (Z=-2.33, p=0.020)** | 0.98 (Z=-0.82, p=0.413) | 0.99 (Z=-0.23, p=0.818) | 1.12 (Z=0.83, p=0.404) |
| Costs of primary care services | 0.81 (Z=-1.62, p=0.106) | **0.98 (Z=-3.30, p=0.001)** | **0.69 (Z=-2.64, p=0.008)** | 0.98 (Z=-0.96, p=0.335) | 0.99 (Z=-0.26, p=0.796) | 1.13 (Z=0.92, p=0.355) |
| Costs of secondary care services | 0.79 (Z=-1.75, p=0.080) | **0.98 (Z=-3.56, p=0.000)** | **0.70 (Z=-2.58, p=0.010)** | 0.97 (Z=-1.03, p=0.305) | 0.99 (Z=-0.27, p=0.786) | 1.12 (Z=0.84, p=0.401) |
| EQ-5D-5L utility | 0.82 (Z=-1.49, p=0.137) | **0.98 (Z=-3.72, p=0.000)** | **0.72 (Z=-2.41, p=0.016)** | 0.98 (Z=-0.71, p=0.476) | 1.00 (Z=-0.09, p=0.926) | 1.12 (Z=0.81, p=0.415) |
| EQ-5D-5L VAS | **0.73 (Z=-2.46, p=0.014)** | **0.98 (Z=-4.02, p=0.000)** | 0.92 (Z=-0.63, p=0.527) | 0.97 (Z=-1.19, p=0.233) | 1.01 (Z=0.36, p=0.718) | 1.28 (Z=1.86, p=0.064) |
| Spending on e-cigarette | 0.92 (Z=-0.62, p=0.533) | **0.99 (Z=-2.72, p=0.007)** | **0.70 (Z=-2.62, p=0.009)** | 0.98 (Z=-0.73, p=0.463) | 1.00 (Z=0.10, p=0.920) | 1.07 (Z=0.53, p=0.598) |
| Spending on NRT | 0.83 (Z=-1.43, p=0.153) | **0.99 (Z=-2.86, p=0.004)** | **0.78 (Z=-1.82, p=0.069)** | 0.98 (Z=-0.93, p=0.354) | 1.01 (Z=0.43, p=0.670) | 1.10 (Z=0.73, p=0.468) |

The respective association between ED sites and reason for ED attendance and missing on 6 months variables was examined by χ^2^ test. The ED site was significantly associated with missing of all follow-up variables while no significant association was found between missing and reason for ED attendance (Table S6).

Table S6 Association between missing at 6 months and ED site and reason for ED attendance in the randomised sample (n=972), examined by χ^2^ test

| **Missing on:** | **ED site** | **Reason for ED attendance** |
| --- | --- | --- |
| Have used NRT | **χ^2^=27.8281, p=0.000** | χ^2^=6.0053, p=0.647 |
| Have been prescribed varenicline | **χ^2^=27.4418, p=0.000** | χ^2^=6.0135, p=0.646 |
| Have been prescribed bupropion | **χ^2^=27.4418, p=0.000** | χ^2^=6.0135, p=0.646 |
| Costs of NRT prescription | **χ^2^=26.4443, p=0.000** | χ^2^=5.8846, p=0.660 |
| Costs of varenicline | **χ^2^=26.4278, p=0.000** | χ^2^=6.1859, p=0.626 |
| Costs of bupropion | **χ^2^=28.7917, p=0.000** | χ^2^=6.0365, p=0.643 |
| Costs of smoking cessation advice | **χ^2^=28.3468, p=0.000** | χ^2^=5.2834, p=0.727 |
| Costs of general practice-based services | **χ^2^=33.0250, p=0.000** | χ^2^=4.5136, p=0.808 |
| Costs of hospital-related services | **χ^2^=32.3563, p=0.000** | χ^2^=4.4348, p=0.816 |
| EQ-5D-5L utility | **χ^2^=34.0562, p=0.000** | χ^2^=5.3046, p=0.725 |
| EQ-5D-5L VAS | **χ^2^=26.6436, p=0.000** | χ^2^=3.5719, p=0.894 |
| Spending on e-cigarette | **χ^2^=22.4556, p=0.000** | χ^2^=4.5687, p=0.803 |
| Spending on NRT | **χ^2^=26.9030, p=0.000** | χ^2^=5.2181, p=0.734 |

The examination of the association between observed values at baseline and missing at 6 months by univariate logistic regression showed that missing on costs of primary care services and costs of secondary care services were associated with costs of healthcare services at baseline (Table S7). The other significant association was found between missing on EQ-5D-5L utility at 6 months and EQ-5D-5L utility at baseline.

Table S7 Association between observed values at baseline and missing at 6 months in the randomised sample (n=972), examined by univariate logistic regression

| **n=972** | **OR (Z, p)** |
| --- | --- |
| **Missing on:** | **Baseline** |
| **6 months** | Have used NRT |
| Have used NRT | 0.67 (Z=-1.78, p=0.075) |
|  | Have been prescribed varenicline |
| Have been prescribed varenicline | 1.03 (Z=-0.05, p=0.963) |
|  | Have been prescribed bupropion |
| Have been prescribed bupropion | Perfect prediction and collinearity |
|  | Have used NRT |
| Costs of NRT prescription | 0.69 (Z=-1.63, p =0.103) |
|  | Have been prescribed varenicline |
| Costs of varenicline | 1.02 (Z=-0.04, p=0.969) |
|  | Have been prescribed bupropion |
| Costs of bupropion | 1.80 (Z=0.41, p=0.679) |
|  | Costs of smoking cessation advice |
| Costs of smoking cessation advice | 0.96 (Z=-1.01, p=0.311) |
|  | Costs of healthcare services |
| Costs of primary care services | **1.00 (Z=2.42, p=0.016)** |
|  | Costs of healthcare services |
| Costs of secondary care services | **1.00 (Z=2.34, p=0.019)** |
|  | EQ-5D-5L utility |
| EQ-5D-5L utility | **0.66 (Z=-2.05, p=0.041)** |
|  | EQ-5D-5L VAS |
| EQ-5D-5L VAS | 1.00 (Z=-1.55, p=0.122) |
|  | Spending on e-cigarette |
| Spending on e-cigarette | 1.00 (Z=0.85, p=0.395) |
|  | Have used NRT |
| Spending on NRT | 0.71 (Z=-1.50, p=0.134) |

The missing values on deprivation index and EQ-5D-5L VAS at baseline were first imputed with their respective pooled mean values in the randomised sample. Missing on reason for ED attendance of randomised patients was coded as ‘unknown’ so as not to be imputed. Missing on reason for ED attendance for accompanying persons was coded as ‘Not applicable’. The missing values on follow-up variables were imputed using chained equation approach. The highest level of missing was found on EQ-5D-5L VAS at 6 months at 45%. The number of multiple imputations was therefore set as 45. The multiple imputation model included the baseline covariates (age, gender, FTCD, if there are other smokers in the household, reason for ED attendance, and ED site), outcome measures at baseline and 6 months (costs of smoking cessation advice, spending on e-cigarette, EQ-5D-5L utility and VAS), costs of healthcare services at baseline, and outcome measures at 6 months (CO-verified abstinence, costs of NRT, costs of varenicline, costs of bupropion, costs of primary care services, costs of secondary care services, and spending on NRT).

Given the large proportion of zero costs, conditional variables (if participant has used NRT, smoking cessation advice, primary care services, secondary care services) at 6 months were originally added to construct a two-step imputation model. The conditional variables were imputed based on baseline variables and then conditioning on them, none-zero missing values were imputed for those with conditional variables as yes. However, when imputing the conditional variables using logit method, it frequently encountered collinearity issue resulting in predictors varying across iterations. Consequently, the conditional variables were removed, and conditional imputation model was dropped. Due to insufficient non-zero observations of costs of varenicline and costs of bupropion, these two variables were imputed but not taken as predictors in other imputations. The outcome variables were imputed, without condition, using predictive mean matching, with the 10 closest neighbouring values to draw from (14). The imputation was performed separately by randomised groups, augmented for perfect prediction.

# Complete case analysis

Table S8 presents the comparison of age, gender and reason for ED attendance between the randomised sample and the complete cases. The complete cases appeared slightly older and consisted of higher proportion of female. Comparing to the randomised sample, complete cases in the control group had lower proportion of those who went to ED for abdominal pain and for injury but higher proportion for other medical issues. However, none of these differences were found to be statistically significant.

Table S8 Comparison of age, gender and reason for ED attendance between all randomised participants and those who had complete outcome measures at 6 months

|  | **All randomised participants** | | **Complete cases** | |
| --- | --- | --- | --- | --- |
|  | **Control (n=488)** | **Intervention (n=484)** | **Control (n=285)** | **Intervention (n=296)** |
|  | **Mean (SD)** | | | |
| Age | 40.5 (13.7) | 40.5 (13.6) | 41.4 (14.0) | 42.0 (13.7) |
|  | **n (%)** | | | |
| Female | 182 (37.6%) | 187 (38.3%) | 123 (43.2%) | 115 (38.9%) |
| Reason for ED attendance | | | | |
| Chest pain | 66 (13.5%) | 54 (11.2%) | 41 (14.4%) | 37 (12.5%) |
| Shortness of breath | 22 (4.5%) | 22 (4.5%) | 13 (4.6%) | 14 (4.7%) |
| Abdominal pain | 40 (8.2%) | 40 (8.3%) | 21 (7.4%) | 24 (8.1%) |
| Injury | 183 (37.5%) | 184 (38.0%) | 95 (33.3%) | 121 (40.9%) |
| Other medical issues | 120 (24.6%) | 122 (25.2%) | 81 (28.4%) | 67 (22.6%) |
| Mental health issues | 10 (2.1%) | 12 (2.5%) | 7 (2.5%) | 7 (2.4%) |
| Other | 40 (8.2%) | 38 (7.9%) | 24 (8.4%) | 18 (6.1%) |
| Missing | 1 (0.2%) | 0 (-) | - | - |
| Not applicable | 6 (1.2%) | 12 (2.5%) | 3 (1.1%) | 8 (2.7%) |

Figure S2 Cost-effectiveness plane and cost-effectiveness acceptability curve of complete case analysis

# Analysis under MNAR assumptions

Table S9 Results of MNAR scenarios 1) and 2)

| Scenario 1) | | Scenario 2) | |
| --- | --- | --- | --- |
| Increased % of imputed costs | Scenario 1) incremental costs | Reduced % of imputed utilities | Scenario 2) incremental QALYs |
| 10% | £24 | 10% | 0.0046 |
| 20% | £16 | 20% | 0.0048 |
| 30% | £9 | 30% | 0.0051 |


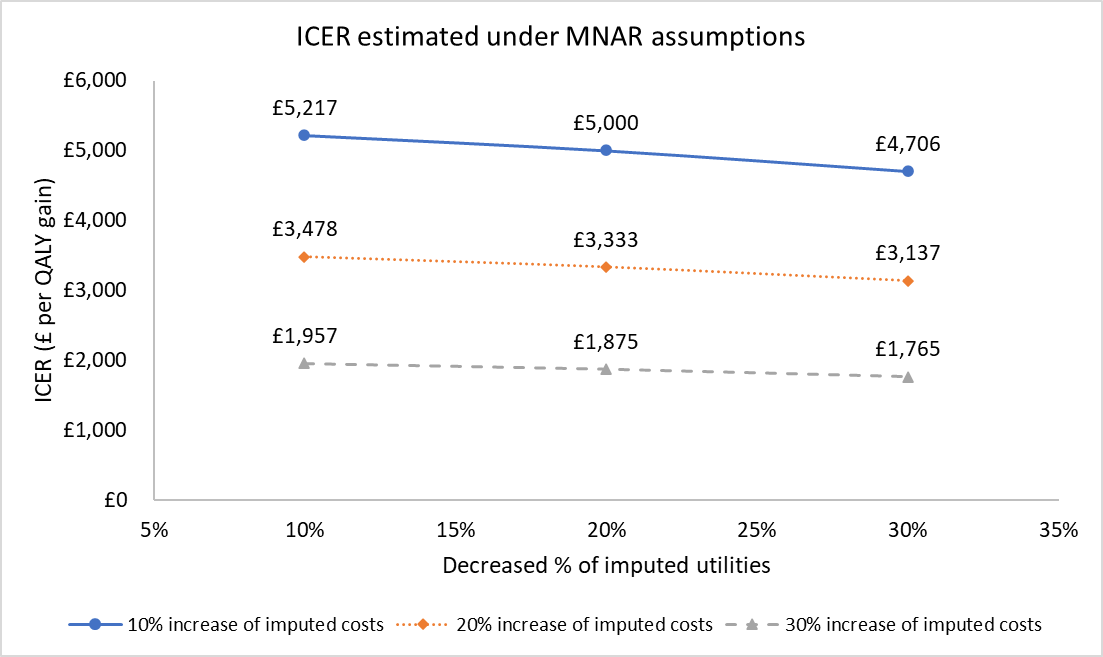


Figure S3 Estimated ICERs under MNAR assumptions

# Participants’ spending on NRT and e-cigarette

Table S10 Participants’' spending on smoking cessation aids over 6 months, by group

| **Spending on** | **Control (n=488)** | **Intervention (n=484)** |
| --- | --- | --- |
|  | **Mean (SE), £** | **Mean (SE), £** |
| E-cigarette | 42 (13) | 82 (9) |
| NRT products | 12 (3) | 9 (3) |
| Total | 54 (14) | 91 (9) |

# Analysis of the broader sample

Figure S4 Cost-effectiveness plane and cost-effectiveness acceptability curve of analysis of the broader sample

# Long-model input parameters

Table S11 Model input parameters estimated from the trial results

|  | **Control** | **Intervention** |
| --- | --- | --- |
| Age (mean) | 40.5 | 40.5 |
| Female (n, %) | 182 (37.6%) | 187 (38.3%) |
|  | **Mean (SE)** | **Mean (SE)** |
| Costs of intervention/usual care | £0.2 (0) | £47.5 (0) |
| Costs of smoking cessation advice | £14 (3) | £11 (3) |
| Costs of NRT products on prescription | £10 (2) | £4 (1) |
| **Costs for abstinence at 6 months in total** | **£24 (4)** | **£63 (4)** |
| **QALYs over 6 months** | **0.290 (0.007)** | **0.303 (0.006)** |
| Abstinence at 6 months |  |  |
| CO-verified | 0.041 (0.009) | 0.072 (0.012) |
| Self-reported | 0.131 (0.015) | 0.252 (0.020) |

# References

1. Vogl M., Wenig C. M., Leidl R., Pokhrel S. Smoking and health-related quality of life in English general population: implications for economic evaluations, BMC public health 2012: 12: 203.

2. Hospital Episode Statistics Analysis, Health and Social Care Information Centre. Hospital Episode Statistics: Accident and Emergency Attendances in England 2014-15; 2016.

3. Royal College of Physicians of London. Tobacco Advisory G., Royal College of Physicians of London. Tobacco Advisory G., Royal College of Physicians of L. Hiding in plain sight : Treating tobacco dependency in the NHS : a report London: Royal College of Physicians; 2018.

4. Department of Health. Reference costs 2015-16; 2016.

5. Jones K., H. W., Birch S., Castelli A., Chalkley M., Dargan A. et al. Unit Costs of Health and Social Care 2022; 2022.

6. Office for national statistics (ONS). Deaths registered in England and Wales: 2021edition; 2021.

7. Doll R., Peto R., Boreham J., Sutherland I. Mortality from cancer in relation to smoking: 50 years observations on British doctors, British journal of cancer 2005: 92: 426-429.

8. Hughes J. R., Peters E. N., Naud S. Relapse to smoking after 1 year of abstinence: a meta-analysis, Addictive behaviors 2008: 33: 1516-1520.

9. Hawkins J., Hollingworth W., Campbell R. Long-term smoking relapse: a study using the british household panel survey, Nicotine & tobacco research : official journal of the Society for Research on Nicotine and Tobacco 2010: 12: 1228-1235.

10. Godfrey C., Parrott S., Coleman T., Pound E. The cost-effectiveness of the English smoking treatment services: evidence from practice, Addiction 2005: 100 Suppl 2: 70-83.

11. National Institute for Health and Care Excellence. NICE health technology evaluations: the manual (PMG36). In: Process and Methods, editor: National Institute for Health and Care Excellence; 2022, p. 196.

12. Wu Q., Gilbody S., Li J., Wang H. I., Parrott S. Long-Term Cost-Effectiveness of Smoking Cessation Interventions in People With Mental Disorders: A Dynamic Decision Analytical Model, Value in health : the journal of the International Society for Pharmacoeconomics and Outcomes Research 2021: 24: 1263-1272.

13. Doll R., Peto R., Boreham J., Sutherland I. Mortality in relation to smoking: 50 years' observations on male British doctors, BMJ 2004: 328: 1519.

14. White I. R., Royston P., Wood A. M. Multiple imputation using chained equations: Issues and guidance for practice, Statistics in medicine 2011: 30: 377-399.
